# Celiac Crisis: A Rare Medical Emergency Case Report in Adult Celiac Disease

**DOI:** 10.1155/crgm/6259846

**Published:** 2025-04-28

**Authors:** Mais Musleh, Amani AlMokbel

**Affiliations:** ^1^Department of Hematology, Faculty of Medicine, Damascus University, Damascus, Syria; ^2^Department of Gastroenterology, Faculty of Medicine, Damascus University, Damascus, Syria

**Keywords:** celiac crisis, celiac disease, pregnant, rare

## Abstract

Celiac crisis (CC) is a rare but potentially life-threatening complication of celiac disease (CD), characterized by severe diarrhea, electrolyte imbalances, and metabolic disturbances. We report the case of a 32-year-old pregnant woman presented significant dehydration, weight loss, and steatorrheic stools. Diagnosis was confirmed by duodenal biopsy, with rapid improvement following a gluten-free diet (GFD) and corticosteroids. The diagnosis of CC was established based on the acute clinical presentation and rapid improvement following a GFD and corticosteroid therapy. This case highlights the importance of early recognition and prompt management of CC, particularly in undiagnosed or untreated CD, to prevent severe maternal and fetal complications.

## 1. Introduction

Celiac disease (CD) is an immune-mediated disorder of the small intestine triggered by dietary gluten in genetically predisposed individuals [1–3]. Although historically associated with pediatric malabsorption, in adults, CD often presents with milder gastrointestinal symptoms or extraintestinal manifestations such as anemia, osteoporosis, chronic fatigue, infertility, or neurological symptoms [2–5].

The prevalence of CD is estimated to be around 1% of the population, but its clinical presentation varies widely, contributing to delayed diagnoses [6]. A rare but severe complication of CD is celiac crisis (CC), characterized by the sudden onset of profuse diarrhea, severe dehydration, hemodynamic instability, and significant electrolyte and metabolic disturbances [7–10]. First described in 1953, CC can be life threatening and requires prompt hospitalization and intensive care. Diagnosis involves recognizing acute malabsorption with positive CD serology and histological confirmation. Management includes immediate nutritional support and strict adherence to a gluten-free diet (GFD).

Despite its seriousness, CC is underreported and not yet included as a distinct entity in most guidelines, including those from the American College of Gastroenterology (ACG) and the European Society for Pediatric Gastroenterology, Hepatology, and Nutrition (ESPGHAN). For instance, the ACG's 2023 guidelines on the diagnosis and management of CD do not mention CC as a separate condition. Similarly, the ESPGHAN guidelines focus on diagnostic criteria and management strategies for CD without addressing CC specifically. This omission underscores the need for heightened clinical awareness and further research into CC [11, 12]. It is increasingly recognized as a severe manifestation of CD, whether as an initial presentation or due to noncompliance with a GFD [13, 14]. This review summarizes the clinical, laboratory, and histological features of CC in adults to provide a clearer understanding of this critical complication.

## 2. Case Report

A 32-year-old female in her third trimester of pregnancy presented to the emergency department with severe dehydration. She reported large volumes of watery diarrhea, occurring 8–10 times daily for the past month, accompanied by a 10 kg weight loss. The stools were watery and fatty (steatorrhea) without blood or mucus. She denied fever, recent travel, medication use, alcohol consumption, or known medical conditions. Clinical examination was unremarkable.

Laboratory investigations revealed severe electrolyte disturbances, including hypokalemia: 2.7 mmol/L (normal: 3.5–5.1 mmol/L), hypocalcemia: 6.9 mg/dL (normal: 8.5–10.2 mg/dL) (corrected calcium level was also assessed), hypophosphatemia: 0.9 mg/dL (normal: 2.5–4.5 mg/dL), and hypomagnesemia: 1.4 mmol/L (normal: 1.7–2.2 mmol/L). Additional findings included hypochromic microcytic anemia (hemoglobin: 9.5 g/dL), hypochromic microcytic anemia: (hemoglobin: 9.5 g/dL) (normal: 12.0–15.5 g/dL), hypoalbuminemia: 2.4 g/dL (normal: 3.5–5.0 g/dL), hypoproteinemia: 4.5 g/dL (normal: 6.4–8.3 g/dL), and iron deficiency: serum iron 23 μg/dL (normal: 50–170 μg/dL). Electrolyte replacement was promptly initiated, leading to modest improvement in ECG abnormalities, including resolution of QT prolongation and attenuation of U waves.

During hospitalization, stool analysis did not identify an infectious etiology, and serological testing for HIV and hepatitis was negative. Liver function tests were within normal limits, and both ultrasound and abdominal x-ray examinations showed no abnormalities.

Due to persistent nonbloody watery diarrhea, significant weight loss, absence of fever, a normal C-reactive protein level, and electrolyte imbalances, an upper endoscopy was performed to confirm the diagnosis. While endoscopy in the third trimester carries inherent risks, it was deemed necessary to establish a definitive diagnosis given the severity of the patient's condition. Endoscopic examination revealed villous atrophy in the duodenum, and biopsies from the duodenal bulb and second portion of the duodenum showed increased intraepithelial lymphocytes (IELs), crypt hyperplasia, and subtotal villous atrophy consistent with Marsh grade 3c.

Serologic testing performed after the biopsy revealed elevated antitissue transglutaminase (anti-TTG) IgA levels at 185 u/mL (positive > 20 u/mL) with normal total IgA 461 mg/dL (normal: 70–400 mg/dL). Although serology is typically obtained before endoscopy, it was performed afterward in this case to further support the histopathological findings.

Fetal wellbeing was closely monitored throughout the patient's hospitalization. Nonstress testing and Doppler ultrasound evaluations revealed no evidence of distress or compromise.

The diagnosis of CD was confirmed based on the clinical presentation, histopathological findings consistent with celiac pathology.

Aggressive electrolyte correction, including phosphate supplementation, was initiated to address severe hypophosphatemia and prevent complications of refeeding syndrome. The patient was started on a GFD, which led to symptomatic improvement within 3 days. Due to the severity of the presentation and delayed response to dietary modification, corticosteroid therapy was initiated after 5 days. The combination of GFD and corticosteroids resulted in complete resolution of diarrhea and paresthesia within 1 week.

Throughout hospitalization, fetal wellbeing was closely monitored via nonstress testing and Doppler ultrasound, which revealed no evidence of distress or compromise. The patient was discharged in stable condition with normalized electrolyte levels and resolution of gastrointestinal symptoms.

Postdischarge evaluations confirmed continued maternal and fetal wellbeing with no neonatal complications. Long-term follow-up is ongoing to monitor potential maternal nutrient deficiencies and ensure optimal fetal growth and development.

## 3. Discussion

CC is a rare but life-threatening complication of CD that necessitates prompt recognition and treatment. Although the incidence of CC is estimated to be under 1% of all CD cases [15], it remains an important consideration in patients presenting with severe diarrhea, significant hypoproteinemia, and profound electrolyte and metabolic disturbances necessitating hospitalization [1–3]. In our case, the patient had severe watery diarrhea accompanied by symptoms of electrolyte imbalance and dehydration without a preceding history of fever [16].

The onset of CC is often associated with poor adherence to a GFD, particularly in patients with a known diagnosis of CD [15–17]. However, as demonstrated in this case, CC can occur in individuals who have not been previously diagnosed, further emphasizing the importance of early recognition. This underscores the necessity for a high degree of clinical suspicion to ensure timely and accurate diagnosis.

A review of the literature indicates that the average age of onset for CC is approximately 53 years, with documented cases ranging from 23 to 83 years [18]. The condition appears to be more prevalent in women than in men, with a reported female-to-male ratio of 15:7 [8, 15, 19, 20]. Common electrolyte and acid–base disturbances associated with CC include severe diarrhea, weight loss, and sometimes hemodynamic instability. Most frequent laboratory alterations reported were metabolic acidosis, anemia, hypocalcaemia, hypokalemia, and hypoalbuminemia, a consequence of the profound malabsorptive state, with our patient presented diarrhea, weight loss, and hypokalemia and hypoalbumania [15, 16].

While endoscopy in the third trimester is associated with risks, it was essential in this case to confirm the diagnosis of CD. Although serology is typically performed before endoscopy, the elevated anti-TTG IgA levels obtained postbiopsy provided additional diagnostic confirmation. The decision to proceed with endoscopy was justified by the severity of the patient's symptoms and the need for definitive histopathological evidence.

Treatment of the patient's CC began with the introduction of a GFD, parenteral fluid replacement, and nutritional support. This approach led to rapid and significant clinical improvement, which is observed in approximately 50% of CC cases. Studies have shown that budesonide may enhance both histological and absorption parameters in CD, potentially by restoring brush border enzyme activity and reducing mucosal inflammation [15, 17].

In our case, treatment involved initiating corticosteroid therapy alongside nutritional support, beginning with a reduced caloric intake of 500 kcal/day (approximately 10 kcal/kg/day) and supplemented with vitamins. This approach led to a favorable clinical outcome. Although refeeding syndrome has not been widely reported in adults with CC, studies indicate a poor prognosis in pediatric cases where refeeding syndrome complicates the condition. The use of corticosteroids, a therapeutic strategy documented in the literature for CC, may worsen the syndrome by further depleting potassium, magnesium, and phosphate levels [18, 21–24].

This case highlights the importance of considering CD in the differential diagnosis of acute diarrhea with severe electrolyte disturbances, particularly in high-risk populations such as pregnant patients. Early diagnosis and individualized management are crucial to prevent complications and ensure favorable maternal and fetal. The combination of GFD and corticosteroids led to a rapid and sustained clinical improvement, underscoring the importance of a tailored therapeutic approach in CC.

In conclusion, CC is associated with significant morbidity and, although rarely described, does occur in adults, often without a clear precipitating factor. Patients presenting with severe, unexplained malabsorption should be evaluated for CD, and treatment with systemic or topical corticosteroids should be considered. Corticosteroids, in conjunction with gluten withdrawal and nutritional support, appear to be beneficial, although symptom resolution may be prolonged. The establishment of formal diagnostic criteria for CC is anticipated to facilitate the identification and management of affected patients.

## Data Availability

Data sharing not applicable to this article as no datasets were generated or analyzed during the current study.
